# Promoting participatory research in chronicity: The ESPRIMO biopsychosocial intervention for young adults with multiple sclerosis

**DOI:** 10.3389/fpsyg.2022.1042234

**Published:** 2022-11-03

**Authors:** Valeria Donisi, Silvia Poli, Maria Angela Mazzi, Francesca Gobbin, Federico Schena, Lidia Del Piccolo, Valeria Bigardi, Alberto Gajofatto, Michela Rimondini

**Affiliations:** ^1^Section of Clinical Psychology, Department of Neuroscience, Biomedicine and Movement Science, University of Verona, Verona, Italy; ^2^Section of Neurology, Department of Neuroscience, Biomedicine and Movement Science, University of Verona, Verona, Italy; ^3^Section of Movement Science, Department of Neuroscience, Biomedicine and Movement Science, University of Verona, Verona, Italy

**Keywords:** clinical psychology and health, multiple sclerosis, co-creation, health related quality of life, participatory research, biopsychosocial (BPS) model, patient engagement, chronic disease

## Abstract

**Background:**

Co-creation allows to develop tailored interventions in chronicity and to increase patients’ engagement. Considering the interacting nature of physical, psychological, and social domains in multiple sclerosis (MS), a biopsychosocial approach to care is crucial.

**Aims:**

This paper aims to present (i) an example of a co-creation process in the context of chronic diseases (ii) preferences and perspectives of young adults with multiple sclerosis (YawMS; aged 18–45) and healthcare professionals (HCPs) on the relevance, objectives, and modalities of a biopsychosocial intervention (named ESPRIMO) and on strategies/barriers to participation.

**Methods:**

A participatory mixed-method approach in three consecutive steps was implemented: online surveys with YawMS (*n* = 121) and HCPs (*n* = 43), online focus groups (FGs) with YawMS, consultation with an advisory board (AB) composed by YawMS, HCPs and researchers. For the survey, descriptive statistics and inductive content analysis have been used for quantitative and qualitative analysis, respectively. FGs and AB were used to deepen the understanding of the survey’s results.

**Results:**

An integrated intervention is extremely relevant according to the perspectives of the main stakeholders. Helping disease acceptance, providing stress management strategies, and supporting emotional expression emerged as the most relevant psychological objectives according to participants. Having tangible benefits, being tailored, and fostering interpersonal relationships emerged as the main preferred characteristics of physical activity. Preferences emerged on the modalities and timing of the intervention, with a venue unrelated to the disease strongly supported. Both HCPs and YawMS highlighted as the most valuable advantages of conducting the intervention online the increased accessibility, while the main limit was the restriction to social interaction (recognized as already limited during the COVID-19 pandemic). Accessibility and lack of time resulted as the main barriers to participation.

**Conclusion:**

The co-creation process gave valuable information on preferences and perspectives of main stakeholders on objectives, modalities, and strategies to improve participation which has been used in the design of the ESPRIMO biopsychosocial intervention. Those results might inform future intervention development in the field of chronicity. The current paper outlined a co-creation methodology which might be replicated in future research on other conditions of vulnerability.

## Introduction

Interventions for patients with chronic disease (CD) are usually developed using a top-down approach: patients are passive users of the intervention that has been designed based on literature evidence and researchers’ experiences and perspectives. Recently participative approaches are becoming more popular and community-academic partnership is becoming a widely accepted methodology in healthcare research and in the design of interventions for health-related quality of life (HRQoL; Bensing et al., 2013). Participatory research can be defined as an umbrella term for research designs and approaches that “use systematic inquiry with the collaboration of those affected by the issue being studied, for purposes of education and taking action or effecting change” (Green et al., 1995). Research partnership is a promising approach that aims to shift the research paradigm from one in which the researcher is the sole expert and the stakeholders are passive subjects of research to one in which researchers and stakeholders collectively integrate their expertise, knowledge and skills (Hoekstra et al., 2020). The role of the stakeholders might vary ranging from the traditional model where the researcher design all the elements of the product, to meta-design where the end-user controls the majority of the process (Leask et al., 2019). Co-creation is in the middle of this continuum and, even if it has not been uniquely defined yet, it can be described as a collaborative generation of knowledge by academics working alongside stakeholders (Greenhalgh et al., 2016). Co-creation is a promising approach for developing tailored intervention in healthcare and it has the advantage to increase adherence and effectiveness (Leask et al., 2019) which represent key elements in the management of chronic conditions. The evidence and best practices about how to implement the co-creation of knowledge and how to involve end-user in health systems are currently being built (Gagliardi et al., 2015; Jull et al., 2017). However, even if co-creation can be achieved using different methodologies (Drahota et al., 2016) and a clear consensus on how to plan and develop co-created research is far from being reached (Leask et al., 2019), the main principle guiding this paradigm are building a relationship between researchers and stakeholders, co-producing knowledge, engaging stakeholder, building resources and fostering support (Hoekstra et al., 2020).

### A biopsychosocial approach to effectively face chronicity

A second core element strictly related to the management and quality of care in chronicity is the adoption of integrated models that recognizes the reciprocal impact and mediating role of different dimensions on illness beside the biological level, such as psychological and social factors. These domains compose the various dimensions that are affected by illness and that can also play a mediating role in the expression of symptomatology and more in general in the acceptance and adaptation to illness. Adjustment to chronicity requires resources in psychological and social domains as well as in the physical/rehabilitative one to maintain and/or promote an adequate HRQoL. Thus, adopting a biopsychosocial approach is highly important (Wade and Halligan, 2017).

However, literature on the development of integrated interventions for CDs targeting all such different domains at the same time seems still scattered. Some efforts have been made on different CDs such as, for example, chronic low back pain (Kamper et al., 2014), stroke care (Kontou et al., 2022) and diabetes mellitus (Suhaimi et al., 2020). More extensive research is needed to tackle the burden of CDs, especially in a neurodegenerative life-limiting chronic condition such as multiple sclerosis (MS), a field in which, to the best of our knowledge, biopsychosocial interventions have not been developed yet.

### The ESPRIMO project: Supporting young adults with multiple sclerosis through a biopsychosocial intervention

MS is usually first diagnosed at the age of 20–40 years (Oh et al., 2018). MS is considered the most common neurological disease that causes disability in young adults (Koch-Henriksen and Sørensen, 2010) with an impact on different personal areas as it interferes with physical (such as gait, vision, and sensory abilities) and cognitive function. Moreover, patients with MS may experience psychological symptoms (such as anxiety and depression; Gajofatto et al., 2019). Having to adapt to a CD with an unpredictable clinical course often have an impact on social dimensions (such as interpersonal relations).

Considering that these aspects interact one with another, a biopsychosocial approach in intervention dedicated to people with MS is needed. With the aim to fill the literature gap on integrated intervention for MS, the ESPRIMO project (Explore, Support, and Promote Resilience In young adults with Multiple sclerOsis) started in 2019 offering a biopsychosocial approach (i.e., ESPRIMO intervention) for young adults with MS (YawMS) aimed at improving HRQoL (Poli et al., 2021; Donisi et al., 2021a,b). The project uses a co-creation approach, thus filling a further gap considering the limited experience with participatory approaches in the MS research field (Giovannetti et al., 2020). The co-creation approach aimed at adjusting and modifying ESPRIMO’s theoretical framework (Donisi et al., 2021a), developed based on a literature review, and to inform the content and the modalities of the intervention based on the experience, preferences and needs of the main MS stakeholders.

Considering this background, the aims of the present article are:

- to present the approaches for the co-creation of a biopsychosocial intervention in the context of chronicity and, in particular, of multiple sclerosis;
- to report the preferences and perspectives of young adults with multiple sclerosis and healthcare professionals on a biopsychosocial intervention.

## Materials and methods

The co-creation was implemented using a participatory mixed-methods research process (Ivankova and Wingo, 2018; DeJonckheere et al., 2019; Olson et al., 2019) including quantitative and qualitative methods and investigating stakeholders’ perspectives, preferences, and suggestions. As part of the project “ESPRIMO,” the present study has been approved by the Ethical Committee of the Verona Hospital (Prog 2676CESC) and registered on ClinicalTrials (NCT04431323). Informed consent was obtained from all subjects involved in the study.

### Participants

Young adults with a diagnosis of multiple sclerosis (YawMS) and healthcare professionals (HCPs) with different backgrounds in healthcare setting were involved in the co-creation of the intervention together with the ESPRIMO team of researchers and clinicians (i.e., neurologists, psychologists, statisticians, neuropsychologists).

An advisory board (AB) has been established at the beginning and consulted throughout the project.

### Procedures

**Figure 1:**
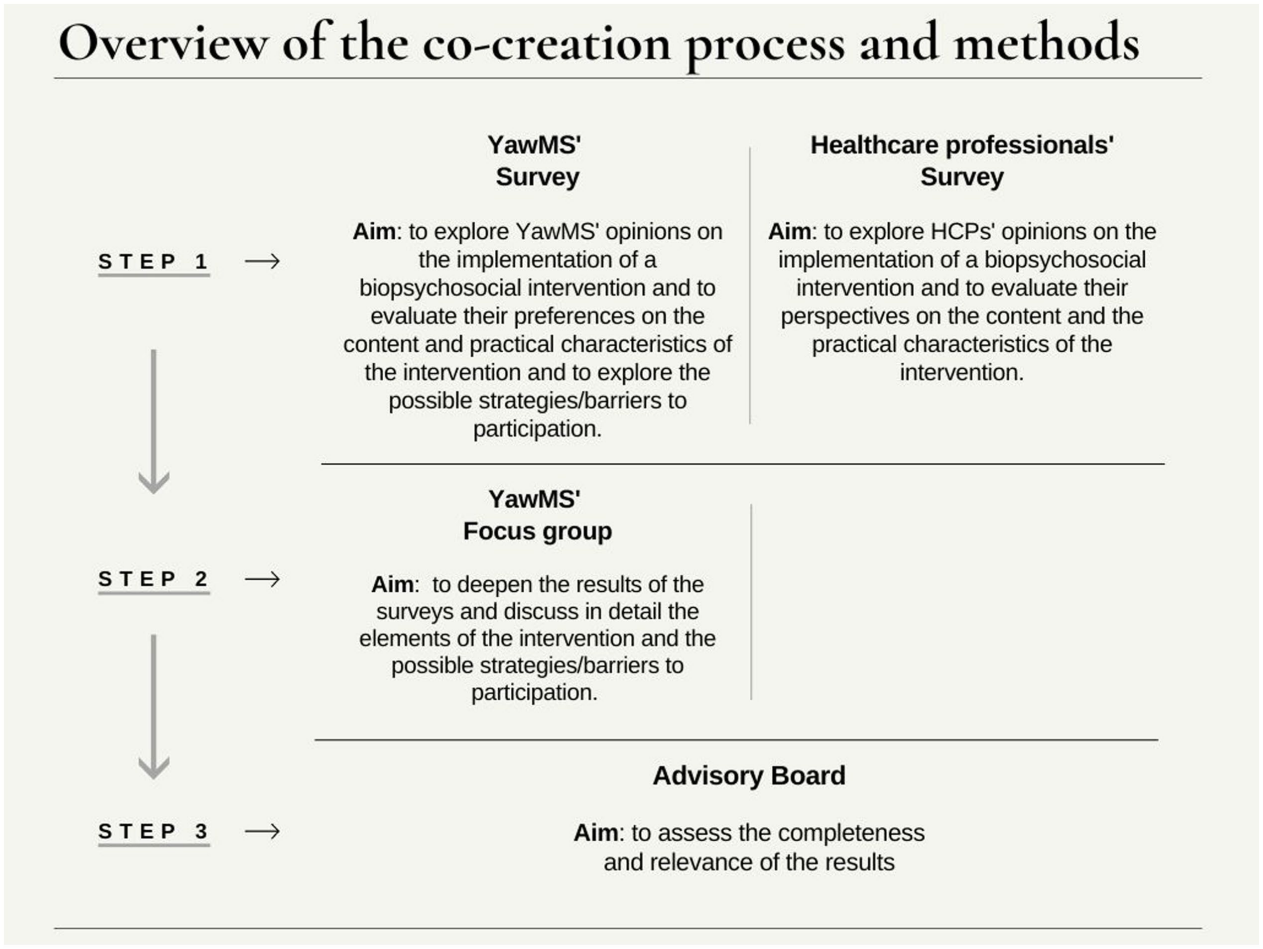
Overview of the co-creation process.

The co-creation consisted of three consecutive steps (Figure 1): surveys, focus groups and consultation with the AB.

#### Step 1: Surveys

Two web-based, anonymous surveys were implemented using the software LimeSurvey and administered between October and December 2020 (Appendix 1).

The first survey was designed for YawMS meeting the following inclusion criteria: age 18–45 years, MS diagnosis, Italian speaker, and electronic informed consent. The survey was composed of closed and open questions divided into three sections aiming to collect: (section one) socio-demographic and clinical information, (section two) patients’ perspectives and preferences about the intervention, and (section three) suggestions about possible barriers/solutions to intervention participation. In section two, participants were asked to rate on a Likert scale ranging from 1 (not important) to 10 (very important) how important is that “an intervention is based on an integrated vision of mind and body” and that “an intervention aims to improve psychological and physical well-being at the same time” and on a Likert scale ranging from 1 (not at all) to 10 (much), and “how much lifestyle can affect the disease.” Participants were then asked to choose a maximum of options among a list (multiple choice with the possibility to give other suggestions if they felt something was missing) about: (i) the main objectives on which an intervention should focus to promote psychological well-being and (ii) the main characteristics of an activity to promote physical well-being Additionally, multiple choice questions with one possible answer were used to investigate the ideal frequency, the ideal venue for psychological and physical activities. Two further open questions explored the limits and advantages of conducting the intervention through online modalities Section three contained two open questions asking about possible barriers to participation and possible solutions.

The same rationale was used to develop the second survey designed for HCPs (Appendix 2), with profession and years of experience with MS collected in the first section and two additional questions (in section two) asking (i) how beneficial an integrated biopsychosocial intervention can be for the care process (on a Likert scale from 1 = not at all to 10 = much) and (ii) the perceived benefits of a biopsychosocial integrated intervention (open question). The following inclusion criteria for HCPs have been considered: being a healthcare professional working with MS patients; Italian speakers; electronic informed consent.

YawMS were recruited using social media (e.g., online groups of YawMS on Facebook, and Instagram), while for HCPs email invitations to take part in the survey were sent *via* email through the MS Hub and SPOKE network of Verona Province with the request of sending the survey to other colleagues (snowball recruitment). Considering that the surveys are part of a larger mixed-method data collection and according to the relevant literature in qualitative research (van Rijnsoever, 2017; Moser and Korstjens, 2018), a sample of at least 50 YawMS and 25 HCPs were estimated sufficient for this step.

#### Step 2: Focus group

The focus group topic guide was developed to gain additional information on preferences and needs regarding psychological and physical activities, and on potential strategies to reduce barriers to participation. Aspects connected to the socio-relational contents of the intervention have been investigated. Two FGs were held in March 2021 and, applying the criteria of data saturation (Onwuegbuzie et al., 2009), were considered sufficient to explore all the relevant topics (Donisi et al., 2021a).

Patients have been recruited at the MS Center of Borgo Roma Hospital in Verona (MS Hub Center, northeast of Italy) by the treating neurologist according to the following inclusion criteria: age 18–45 years, MS diagnosis, Italian speakers, and electronic informed consent. Before participating, the participants have to fill out a brief online questionnaire to collect socio-demographic and clinical information. Focus groups have been conducted online (using Zoom) and video recorded; after the completion recordings have been transcribed *verbatim.*

#### Step 3: Consultation with the advisory board

The Advisory Board is composed of a clinical psychologist, a neurologist, a movement scientist, a health sociologist and four YawMS.

The presentation of the ESPRIMO project and the results of the quantitative and qualitative parts to the AB were held online by the ESPRIMO researchers. Contradictory results from the surveys and the FGs or further suggestions or critical points raised by the AB were examined in an open discussion. One researcher of the team took notes of the exchanges. A final report was sent to all the members of the AB to check for completeness and correctness of the data collected.

### Data analysis

The quantitate results of the survey have been analyzed using descriptive statistics. An inductive content analysis has been applied to analyze the qualitative results of the open questions. In particular, the participants’ answers have been reported in an Excel file, and two researchers have analyzed the text and elaborated possible labels in line with the guidelines (Moretti et al., 2011). As a final step, all answers have been coded using the finalized labels, and the frequency distributions have been calculated.

Results of the FGs and the advisory board were used to deepen the understanding of the survey’s results and the information on the relevant topic. Each main topic was checked against the results of the survey to see if there was agreement or not and to highlight specifications.

## Results

### Step 1: Surveys

The surveys reached a sample of 121 YawMS and 43 HCPs. The majority of YawMS who responded were female and had a (self-reported) diagnosis of relapsing–remitting multiple sclerosis; the mean age was 33 ± 7 years.

Neurologists (53%), psychologists (23%), physiatrists (14%), physiotherapists (5%), and nurses (5%) filled out the survey dedicated to HCPs; the mean age was 40 ± 10 years.

Appendix 3 presents detailed characteristics of the participants, while the survey results have been reported in the following paragraphs.

#### Relevance and usefulness of a biopsychosocial intervention

Most of the participants thought that an intervention based on an integrated mind–body vision is extremely relevant (YawMS: mean 8.9 ± 1.3; HCPs: mean 8.7 ± 1.6), and that is highly important to simultaneously improve psychological and physical well-being (YawMS: mean 9.3 ± 1.1; HCPs: mean 8.9 ± 1.4; Table 1).

**Table 1:** Survey with young adults with MS and with healthcare professionals: opinions regarding relevance and usefulness of a bio-psycho-social intervention.

Questions	YawMS				Healthcare professionals			
	*n*	mean	SD	range	*n*	mean	SD	range
How important is it that an intervention is based on an integrated vision of mind and body?	101	8.9	1.3	5–10	43	8.7	1.7	4–10
How important is it for an intervention to aim to improve psychological well-being and physical well-being at the same time?	102	9.3	1.1	5–10	43	8.9	1.4	5–10
How much can lifestyle affect the disease?	102	7.7	1.9	1–10	43	7.7	1.5	3–10
How beneficial an integrated biopsychosocial intervention can be for the care process?^*^					43	8.4	1.6	3–10

* Question included only in the survey for HCPs.

When specifically asked how beneficial an integrated biopsychosocial intervention can be for the care process, HCPs answered positively (mean 8.4 ± 1.6). Moreover, the following perceived benefits were added: *benefits in the care path* (*N* = 17), *psychological benefits* (*N* = 14), *adjustment to the disease* (*N* = 7), *social benefits* (*N* = 7), *global benefits* (*N* = 6), and *physical benefits* (*N* = 2).

As regards the *benefits in the care path,* HCPs hypothesized an improved treatment adherence, more realistic expectations regarding treatments, and enhanced HCPs-patients relationships and trust. In the words of a participant “*putting the person at the center with their specific physical, emotional, cultural, and social characteristics allows the doctor to better understand their needs and to establish a more effective care relationship*.” Regarding the *adjustment to the disease*, HCPs highlighted that an integrated intervention might foster a better understanding of the symptoms, an acceptance of MS and promote a more favorable concept of disability also improving its acceptance within the family. According to HCPs, an integrated intervention might reduce the risk of relapses (*physical benefits*) and could improve interpersonal relationships and foster social inclusion and stigma reduction (*social benefits*). *Global benefits* are also expected (e.g., a better quality of life). As far as *psychological benefits*, HCPs considered an improvement in mood and self-efficacy and reduction of stress, increased awareness of one’s functioning, increased ability to cope with difficulties and disability, all of which might also have a positive impact on relapses.

#### Psychological objective of the intervention

Regarding the main objectives to promote psychological well-being, among the listed options the most rated both by YawMS and HCPs were helping to accept the disease and providing stress management strategies. The third most rated by YawMS was supporting the expression of emotions and concerns, while the HCPs indicated “increase self-efficacy in managing the disease” (Table 2).

**Table 2:** Survey with young adults with MS (*N* = 121) and with healthcare professionals (*N* = 43): needs and preferences regarding the main objectives of the psychological activity and the characteristic of the physical activity.

	YawMS	HCPs
**a) In your opinion, in order to promote PSYCHOLOGICAL well-being, what are the main objectives on which an intervention should focus? (indicate a maximum of four objectives)**		
List of possible answers	Responses, *n* (%)	Responses, *n* (%)
It should help me to accept the disease and its consequences	72 (18.9)	29 (17.9)
It should provide me with advice and stress management strategies	68 (17.8)	29 (17.9)
It should help me to express my emotions and concerns	51 (13.4)	22 (13.6)
It should change the way I see things	48 (12.6)	13 (8)
It should increase my sense of self-efficacy in managing the disease	37 (9.7)	29 (17.9)
It should make me aware of my emotions	35 (9.2)	14 (8.6)
It should motivate me to change	28 (7.3)	14 (8.6)
It should help me to process past traumas	24 (6.3)	0 (0)
It should inform me about the risks of an unhealthy lifestyle	18 (4.7)	12 (7.4)
**b) In your opinion, what characteristics should the proposed physical activity have in order to promote PHYSICAL well-being? (indicate a maximum of four characteristics)**		
List of possible answers	Responses, *n* (%)	Responses, *n* (%)
It should have tangible benefits for the body	58 (29.3)	10 (9)
It should be adapted to my physical needs	52 (26.3)	34 (30.6)
It should encourage me to continue with physical activity even after the intervention	40 (20.2)	29 (26.1)
It should let me get to know new people	25 (12.6)	26 (23.4)
It should be fun	16 (8.1)	7 (6.3)
It should teach me something new	5 (2.5)	3 (2.7)
It should be a new activity	2 (1)	2 (1.8)

Thirty-one YawMS indicated other potentially relevant objectives; the following are the main categories emerged: increase independence, manage anger and negative emotions, develop a positive approach, help to go back to normal life, work on self-esteem, coping with difficulties, receiving support in a delicate period of time (e.g., motherhood), handling the psychological effects of taking medications, understanding how to communicate the diagnosis and how to react to judgment, dealing with stigma and feelings of being a burden, sharing experiences with others YawMS.

#### Physical objective of the intervention

Regarding the preference on the main characteristics of an activity to promote physical well-being, having tangible benefits on the body, being tailored to each individual’s physical needs, fostering relationships with others YawMS and encouraging to continue after the intervention were reported more frequently (Table 2).

Other aspects that were added by YawMS (*N* = 17) included: becoming independent, improving balance, and endurance, strengthening the muscles and resistance to fatigue; experimenting dynamic activities; reducing stress. Additionally, one HCP stated that the intervention should improve the perception of one’s body, its potentialities, and its limits.

#### Intervention modalities and timing

As regards practical aspects of the intervention (Table 3) the majority of YawMS preferred one or two meetings per week and a neutral venue not connected to the disease (both for the psychological and physical activities).

**Table 3:** Survey with young adults with MS: needs and preferences regarding intervention modalities and timing.

Questions	Levels	*N* (%)
In your opinion, what would be the ideal frequency for meetings?	Two meetings per week	41 (40.2)
	One meeting per week	41 (40.2)
	One meeting per month	3 (2.9)
	One meeting every other week	17 (16.7)
In your opinion, what would be the ideal venue for meetings related to psychological aspects?	My hospital or treatment center	26 (25.5)
	*Via* telematics (using a videoconferencing platform, e.g., Zoom, skype)	15 (14.7)
	The seat of a patient association	18 (17.6)
	A neutral place not connected to my illness (e.g., gym, social club)	43 (42.2)
In your opinion, what would be the ideal venue for meetings relate to physical activity?	My hospital or treatment center	19 (18.6)
	*Via* telematics (using a videoconferencing platform, e.g., Zoom, skype)	4 (3.9)
	The seat of a patient association	12 (11.8)
	A neutral place not connected to my illness (e.g., gym, social club)	67 (65.7)

Out of 121 participants in the survey, 102 answered to these questions.

Two open questions focused on online delivery (Table 4). The most frequent advantage, both for YawMS and HCPs, of conducting the intervention online would be increased accessibility which might result in higher participation. According to responders, online modalities could lower COVID-19 related risks. The most frequently reported downside of online modalities, both for HCP and YawMS, is the limitation to interpersonal relationships (see more details in Table 4).

**Table 4:** Survey with YawMS (*N* = 121) and with HCPs (*N* = 43): qualitative analysis of answer regarding limits and advantages of conducting the intervention through online modalities.

Advantages of online modalities				Limits of online modalities			
Theme	Advantages labels	Frequency		Theme	Limits labels	Frequency	
		*YawMS*	*HCPs*			*YawMS*	*HCPs*
*Accessibility*	To reach a wider public; Lower costs for participants; Easiness of participation as there is no need to move or to relay on caregivers; More easily fitted in one own’s agenda not having to travel losing time and having the possibility to connect anywhere	28	24				
*COVID-19 pandemic*	Lower the risk of getting infected (higher safety); Intervention guaranteed even during lockdowns;	8	3				
*Organizational aspects*	Easiness of organization and management for HCPs	/	3				
				*Interpersonal relationships with other participants*	Online interactions are different from in-persons and might prevent or limit social connections; Unnatural or difficult communication with lack of paraverbal aspects; Limitation of informal aspects of sociability; Feeling of isolation	30	19
				*Limited interactions with HCPs*	Limited possibility of motor correction and manipulation; Lower connection and empathy	10	5
*Facilitation of expression*	Higher comfort in expressing oneself or doing physical activities, overcoming their shyness, anxiety or embarrassment	5	4	*Limitation in expression*	Lower genuineness and comfort in expressing oneself; Lack of privacy and shame of being heard	10	/
*Technical advantages*	Makes it easy and practical to participates and might give the possibility of recording encounters	1	/	*Technical downsizes*	Costs; Possible malfunctions; Low familiarity with digital tools; Easiness of distraction	6	7
*Higher motivation*	Higher compliance to the intervention	/	2	*Lower motivation*	Lower motivation in taking part in the intervention; Less involvement and engagement	5	6
None^*^		19	2	None^*^		5	1
	Total^**^	64	40			68	39

Please note that limits and advantages have been reported in the same line when they represented conflicting views on the same topic.

* Participants’ answers were not relevant to the theme and thus were not categorized in labels, or participant wrote “no answer.”

** Some participants reported more than one label.

#### Barriers and solutions to foster participation

Table 5 shows the themes, a brief description for specific labels and their frequencies for both YawMS and HCPs on the possible barriers and solutions to foster participation to the intervention. Participants mostly reported accessibility as the main drawback, including distance from the venue, lack of public transport and being dependent on a caregiver for coming to the encounters. As examples of possible solutions to increase accessibility, YawMS and HCPs proposed holding encounters online or selecting an easily accessible venue.

**Table 5:** Survey with YawMS (*N* = 121) and with HCPs (*N* = 43): qualitative analysis of barriers for patients to participate in the intervention and possible solutions.

Barrier theme	Barrier labels	Frequency		Possible solutions labels		Frequency	
		YawMS	HCPs			YawMS	HCPs
Accessibility barriers	The venue might be far from home or difficult to reach with public transport; Some participants might not be independent; Fatigue or presence of physical symptoms	21	19	Using online tools, offering intervention at the patients’ house, or transport solutions, offering the intervention in different venues, granting possibility to skip some encounter, choosing a venue suitable for the whole group to lessen the distance; Help patients to accept and overcome fatigue		19	9
*Example of quote by the participants*	*“If the encounters are held far from home, because sometimes I cannot drive for many miles, sometimes I have problems with one eye and while I’m driving it hurts and I have to stop”* [by YawMS]			*“Identification or creation of several structures that are able to offer sports motor activities for people with disabilities, guaranteeing diversified time slots, specific equipment and customization of the proposals”* [by HCP]			
Psychological barriers	Difficulties in talking with others; Fear of judgment from other people, of not being understood, of seeing other people with greater disability, of failure and belief of not being adequate; Low motivation; Not seeing the benefit of a psychological approach	13	22	Proposing activities that are new and entertaining, fostering a welcoming and non-judgmental environment, normalizing symptoms; Helping awareness of one own’s emotions, using motivational interview, offering psychological support		3	11
*Example of quote by the participants*	*“Fear of encountering more serious situations than theirs, and consequently anticipating their own worsening and experiencing a form of threat”* [by HCP]			*“Underline in the communication that the environment is welcoming and non-judgmental”* [by YawMS]; *“Show the feasibility by everyone regardless of their skills*” [by YawMS]			
Characteristics of the intervention	Not having enough time to take part in the encounters due work, family, or personal reason; People in the group might have different ages or different experiences (e.g., different diagnosis)	16	17	Offering encounters later on the evenings or on the weekend, offering different, tailored, and flexible timeframes; Creating small groups with similar ages and physical abilities		9	17
*Example of quote by the participants*	*“The time to dedicate to it that you have to cut out from other work-related or non-work-related activities*” [by YawMS]			*“Try to detect, through an online questionnaire, what are the difficulties and needs and in which time range they are available”* [by HCP]			
No barriers^*^		**11**	**1**	No solutions^*^		**14**	**4**
	Total^**^	**61**	**59**			**45**	**41**

* Participants’ answers were not relevant to the theme and thus were not categorized in labels, or participant wrote “no answer.”

** Some participants reported more than one label.

The other most frequent theme regarded psychological barriers. As a possible solution, creating a positive environment was highlighted together with emotional support. Finally, other barriers regarded the practical characteristics of the intervention. Possible solutions reported by participants regards the tailoring of the intervention characteristics to the participants’ needs.

### Step 2: Focus groups

A total of 31 YawMS took part in the two focus groups (mean age of 32.8 ± 6.6; female 71%; 92.3% diagnosis of relapsing–remitting MS). No YawMS participating in the FGs had significant limitations, while 23% had partial limitations, and 77% had no limitations.

#### Psychological objective of the intervention

Regarding the main psychological objectives of the intervention, all participants agreed on the ones most frequently reported in the surveys. Regarding the acceptance of the disease, receiving practical suggestions, for example, on how to deal with a specific symptom or with uncertainty, should be considered in the delivery of the intervention. Having to deal with the disease made YawMS realize their functioning and other difficulties because the disease is connected to all areas of their lives: accepting the disease is the first step that also helps to intervene on other aspects (“*And then [after the diagnosis]at that point many things come out, in short: about you, how you are, what you do, how you act in certain situations and moments or in the workplace*).” As an adjunction, single comments regarded that it is important to: “*let things emerge and think about them*” not to be overwhelmed; increase motivation to act positive change; improve self-efficacy in the management of the disease, because of his personal difficulties in adapting to a new lifestyle.

#### Physical objective of the intervention

Participants agreed on the main characteristics that emerged in the survey regarding physical activity. For example, a participant stated: “*If we decide to take a walk, [it is important] that I can do it, that I can have fun and that I am able to repeat it even alone and that it is good for me*.”

In addition, getting to know people dealing with the same condition and doing physical activities together has been considered a way of increasing motivation. Interaction with other people and music are a way, according to participants, to have fun while also doing something useful for the body (“*Doing [physical activity] alone I think that everyone is a little less encouraged*”; “*Also that there is a bit of fun and maybe even music*”). Dance has been considered a good way to mix all these elements, also helping to create social connections and prevent social isolation linked to diagnosis. Moreover, dance might help to express oneself in a more effective way, “*allowing to express even emotions*.” Nevertheless each individual impairment such as balance should be acknowledged, and one participant suggested to offer different types of dances. One participant cited the possibility to organize evening events based on dance activities.

#### Socio-relational aspects of the intervention

Participants stated that sharing the common experience of living with MS can foster a feeling of comfort, for example, a participant stated: “*In my opinion, the fact that we are all on the same boat puts us a little more at ease[…] because we all have a lot of things in common on this*.” One participant highlighted that after diagnosis people might isolate themselves and, therefore, having the possibility to share concerns and anxieties with people in a similar condition might increase the sense of belongingness.

Working on oneself to acquire social skills and strategies to interact with friends, family, or colleagues has also been cited by participants as a relevant objective in socio-relational domain. Moreover, the discussion focused on the need to sensibilize the general public about the disease using leaflets or designing specific events.

As an additional topic of the FGs, participants discussed whether if, during the physical or phycological activities, they would like to bring friends or family members. In the first focus group, people expressed the wish to have this opportunity to connect different social microcosmos and that this could be a way to let beloved ones see other experiences. However, in the second focus group, all participants agreed that they would prefer not to be accompanied and that, if this might be the case, all patients taking part in the intervention should consent. In fact, the presence of other people without MS might create discomfort in the group. Moreover, referring to their own experience, participants stated that the relationship with their beloved might change in unexpected ways after participating in such encounters and they would not be comfortable in inviting friends. Nevertheless, participants in both groups expressed the wish to be accompanied by a partner or by some member of the family in some dedicated informal encounters.

#### Intervention modalities and timing

The survey results on the modalities and timing of the intervention have been discussed during the FG, with extensive considerations regarding the barriers and advantages of the online modalities versus the in-person ones. The main contents have been summarized in Table 6.

**Table 6:** Synthesis of the focus group with YawMS in comparison with the main results of the survey: needs and preferences regarding intervention timing, venue, and modalities.

Topics	Survey main results	Focus group specifications and additional considerations
The ideal frequency	One or two meetings per week	In partial agreement with the survey, participants believed that holding one encounter per week would be a good way to foster participation. In fact, two encounters a week, even if considered useful and appealing might be unsustainable because of having to balance work, family, and personal time (*“Surely one meeting, which I would be able to keep up with, will be less binding; not because I do not like two encounters, but it [one meeting] would be more sustainable”*). Moreover, few people might have difficulties also with one encounter per week (*“For me, even one meeting a week is not exactly that simple”*) with one encounter every other week has been also suggested for the timing of the intervention. Moreover, participants suggested to choose a time in the day during which public transportations are available and one might be more available *“*[the intervention] *could be implemented at a time of the day when there is public transport available.”*
The ideal venue	A higher percentage of respondents prefer a neutral venue	All participants agreed, in line with the results of the surveys, that a neutral venue not connected to the disease is preferable. This would help to lower the burden of the disease as MS would not be perceived as the only identifying characteristic of the group, but it would be one of the reasons why people meet at the encounters. Moreover, participants suggested a place connected with nature (*“Maybe it can be thought of trying to see each other outdoors, perhaps in a park”*) as it has been suggested to be a good way to relax. According to participants the chosen venue should be easily accessible and served by public transport
The online modalities	A low percentage of respondent prefer the online modalities	Participants confirmed the limits and advantages of conducting the intervention online emerged during the surveys. In fact, the most cited advantage was accessibility as people far away might take part in the intervention and even shy people might be more prone to participate. Moreover, being at home might increase the feeling of comfort (*“It’s my home so I feel at ease”*). As the main limit, online modalities might inhibit informal social interactions because *“It misses the physical aspects of getting to know people.”* A participant stated that *“In my opinion the online modalities, if you really cannot do without it, that’s fine, it is better than nothing However, if I were to talk about multiple sclerosis and had to do activities with these people, I would feel better seeing them in person.”*
	Different limits and advantages of conducting the intervention online emerged without a conclusive preference	As a possible solution to increase social interaction, one participant suggested to organize small group activities during online encounters. One person highlighted that since the beginning of the pandemic all activities have been delivered online through smartphones or computers and this modality is becoming tiresome (“*Given that I am tired of online modalities, because I have practically lived in front of my PC for a year for various reasons”*). Some participants stated that, being personal protective equipment available, there is no need to think of an online intervention to prevent the spread of the COVID-19 while two participants said they might feel unsafe or anxious to do group physical activities indoor (*“It happened to me in recent days, of meeting people, of taking all the precautions, but in any case I was hardly able to relax”*). Moreover, face masks, might hamper communication and emotional expression, however a potential solution (as suggested even above regarding venue) could be to meet outdoors.

#### Barriers and solutions to foster participation

Regarding barriers to participation, as emerged in the surveys, participants in the FGs highlighted that people might find it difficult to have time to take part in the intervention due to work or family commitments. A theme that did not emerged during the survey is the possible interference of therapies with the participation in the intervention during the pandemic: “*Because maybe someone takes immunosuppressants with very low immune defences […]perhaps it is not so convenient to move*.”

### Step 3: Advisory board

The involvement of YaMS during all phases of the project through a co-creation approach has been appreciated by the Advisory Board. Some specific comments that emerged during the discussion highlighted the strengths of the project and offered ideas for improvement.

In general, the AB confirmed previous results on the usefulness of an intervention that takes into consideration the biopsychosocial domains at the same time. Dance, supported in the FG as a possible ESPRIMO physical activity, was considered positive highlighting the value of music in fostering relationships and improving the enjoyability of the activity. According to the expert in movement sciences some elements of music such as rhythm might enhance motor gains.

The duration of the sessions was discussed along with the suggestion to provide for an additional “booster” session 1 month after the end of the intervention to allow consolidation of the improvements.

The attention to social aspects, has been considered fundamental, as it builds connectedness and allows to share experiences between YawMS: a key element in the process of adjusting to the disease. Regarding the controversial results on online and in-person modalities, the AB stressed the importance of contemplating in-person activity which has been considered the most appropriate way to foster involvement and connectedness, also considering the reduction of social activities due to the COVID-19 pandemic.

## Discussion

### The relevance of the co-creation process

The co-creation process gave valuable information about stakeholders’ perspectives and preferences for the development of a biopsychosocial intervention in the field of MS. Different consecutive steps have been used (surveys, FGs and the AB) in order to enrich and deepen the data collected.

Involving patients in research and valuing their opinion as experts can inform the design of services starting from the experience and needs of people that will use them (Morote et al., 2020). Programs that are designed based on the patients’ preferences are more sustainable for participants and more sensitive to the specific context, thus possibly reducing the dropout rate (Lo and Karnon, 2019). The co-creation process has been introduced as a particularly valuable strategy in the current paper for at least two reasons: the particular target of the ESPRIMO intervention (i.e., young adults with MS) and the specific contextual conditions in which ESPRIMO has been developed. Indeed, as a first reason, a diagnosis of MS at this age might be intertwined with the personal and interpersonal goals typical of this phase of life, creating peculiar needs. Accordingly, for example, many barriers to participation discussed in the survey and focus groups results regard the overlap of the intervention time with the daily activities typical of the age. Secondly, the participatory methodology is particularly essential in this historical period of the COVID-19 pandemic that has brought changes and challenges and potentially created new psycho-social needs, especially for young people and for patients with medical frailties (Xiong et al., 2020). Indeed, in a previous Italian study targeting YawMS in the aftermath of the COVID-19 pandemic, psychological distress (even in relation to the change that occurred in treatment and healthcare services) and the need for psychological interventions emerged (Donisi et al., 2021b). As well as reported by participants in the co-creation, during the pandemic, people had to use online tools to socialize and reduce loneliness, both because of government restrictions to social contact both to prevent possible contagion. However, using digital technologies changes the way we connect and interact (Shah et al., 2020); for this reason, understanding social needs and preferences regarding the choice of online or face-to-face modalities was especially useful in the current paper.

### The confirmed value of the biopsychosocial approach from the main stakeholders’ perspective

The value of the biopsychosocial approach was confirmed by results throughout all steps of the co-creation, with a clear recommendation, as all parties involved might benefit from it. Specifically, the responses of YawMS and HCPs highlighted the importance of developing interventions using an integrated vision of mind and body, that simultaneously target physical and psychological well-being. HCPs highlighted possible benefits for patients, for the course of the disease and for the general process of care, including the improvement of doctor-patient relationships which represent a valuable aspect according to the literature (Price et al., 2021).

Moreover, the results suggest that each activity should not only target one specific component but that the bio-psycho-social domains are highly integrated. For example, some answers regarding physical activity encompassed themes pertaining social or psychological domains. In fact, according to participants, physical activity should also aim to reduce stress and foster relationships with others YawMS, once again reinforcing the importance of a biopsychosocial approach in the design of the intervention. However, the interaction between different components must be acknowledged all throughout the intervention design and should be targeted as a specific aim and should not be just a collateral result.

### Preferences and perspectives on the psychological, physical, and social intervention domains

Regarding the psychological domain, the main objectives highlighted by YawMS, and also supported by HCPs, regarded acceptance of the disease, stress management and emotional expression. This is in line with previous results in which YawMS stressed the importance of reducing unpleasant emotions and promoting strategies to accept MS as the most relevant aim of a psychological intervention (Donisi et al., 2021b) and with the relevance of acceptance principles in the psychological literature in the chronicity field (Giovannetti et al., 2021). People who accept their own illnesses are more optimistic, experience fewer negative emotions related to the disease, and have a higher rate of adherence to recommended treatments (Dymecka et al., 2021; Kołtuniuk and Rosińczuk, 2021). Interestingly, participants added other objectives that they considered important; however, those answers might be seen as more specific topics of the three main reported themes. During the focus group, participants reinforced the importance of working on MS acceptance, also focusing on the relationship between disease and other areas of life. Obtaining these specifications during the co-creation steps was useful for defining the specific contents of the intervention and enriching the examples in the materials proposed.

Regarding the physical activity domain, the main characteristics, according to stakeholders, are that it should have tangible benefits on the body, be tailored to each individual’s physical needs, and encourage to continue after the intervention. During the focus group, the main results of the survey in the area of physical activity were confirmed, such as the relevance of an activity that should be beneficial and adapted to the level of the individual. In line with the literature in this field (Wiersma, 2001; El-Sherif, 2016) participants in the FGs prioritized the role of pleasantness, highlighting that doing physical activities together with other YawMS is a way to increase pleasantness and motivation. Dance has been considered a good way to socialize (also offering opportunities to organize events for a broader community) and to incorporate pleasant elements such as music; which is in line with the existing literature on dance activities in MS (Salgado and de Paula Vasconcelos, 2010; Mandelbaum et al., 2016; Ng et al., 2019; Van Geel et al., 2020).

Regarding the socio-relational domain of the intervention, doing the intervention with other young people that share the experience of living with MS emerged as a positive element that can help people to prevent social isolation and to foster meaningful interaction with alike people. Having to share a path with people that can easily comprehend the struggles of living with MS can help to build a positive environment, however, the broader social context should not be forgotten. Indeed, participants highlighted the need to learn specific skills to interact with their personal friends, family, or colleagues and the importance of diminishing stigma-related aspects in the general population. In fact, stigma is a social factor that can affect mental health in MS; however social resources (e.g., social support, sense of belonging) might protect people from stigma’s negative consequences (Cadden et al., 2018).

### Preferences and perspectives on modalities and timing of the intervention and on fostering participation

The co-creation phase made it possible to inform the choice of the intervention on practical aspects which are fundamental to maximize the results and the feasibility of the intervention.

Regarding the timing, the intervention should have modalities that have low interference on the person’s life and be sustainable while also being pleasant and enjoyable for the person. This is particularly relevant considering the target population (young adults) that often works or studies, has low levels of disability and has an active personal (e.g., hobbies and personal interests) and social life (e.g., friends, family) potentially making them busier and also already involved in other psychological, physical or social activities. In general, an intervention dedicated to young adults should fit in their busy daily schedule and should consider strategies to reduce psychological barriers to participation, including different types of fear in interacting with other people (e.g., judgment, not being understood, failure, seeing other people with greater disability).

Linked to the previous aspect, different insights on the limits and advantages of conducting the intervention online emerged and could be considered in future research in this field. To sum up, online modalities could be accessible to a wider public and could lower the risk of COVID-19 infections; however, reduced sense of belonging and social connections (which might be relevant in the aftermath of social restriction during the first year of the pandemic—during which this research was held) and increased feelings of isolation have been stressed as the main limits of this modality. Some aspects regarding the facilitation/limitation in personal expression, the level of motivation and the technical aspects of the online modalities were more controversial; however, a slight prevalence of negative considerations.

In-person encounters should also consider an easily accessible venue. The relevance of a non-medical venue for the intervention was strongly recommended by YawMS. Recent literature supports this suggestion; for example, an external de-medicalized venue was preferred for physical intervention for stroke patients (Young et al., 2021).

### An overview of the ESPRIMO intervention: A summary of the co-creation outcome

Based on the suggestions regarding possible barriers to participation, the ESPRIMO program has been designed to maximize the effects on wellbeing while also considering accessibility and people’s difficulties in participating in too many encounters due to personal reasons.

**Figure 2:**
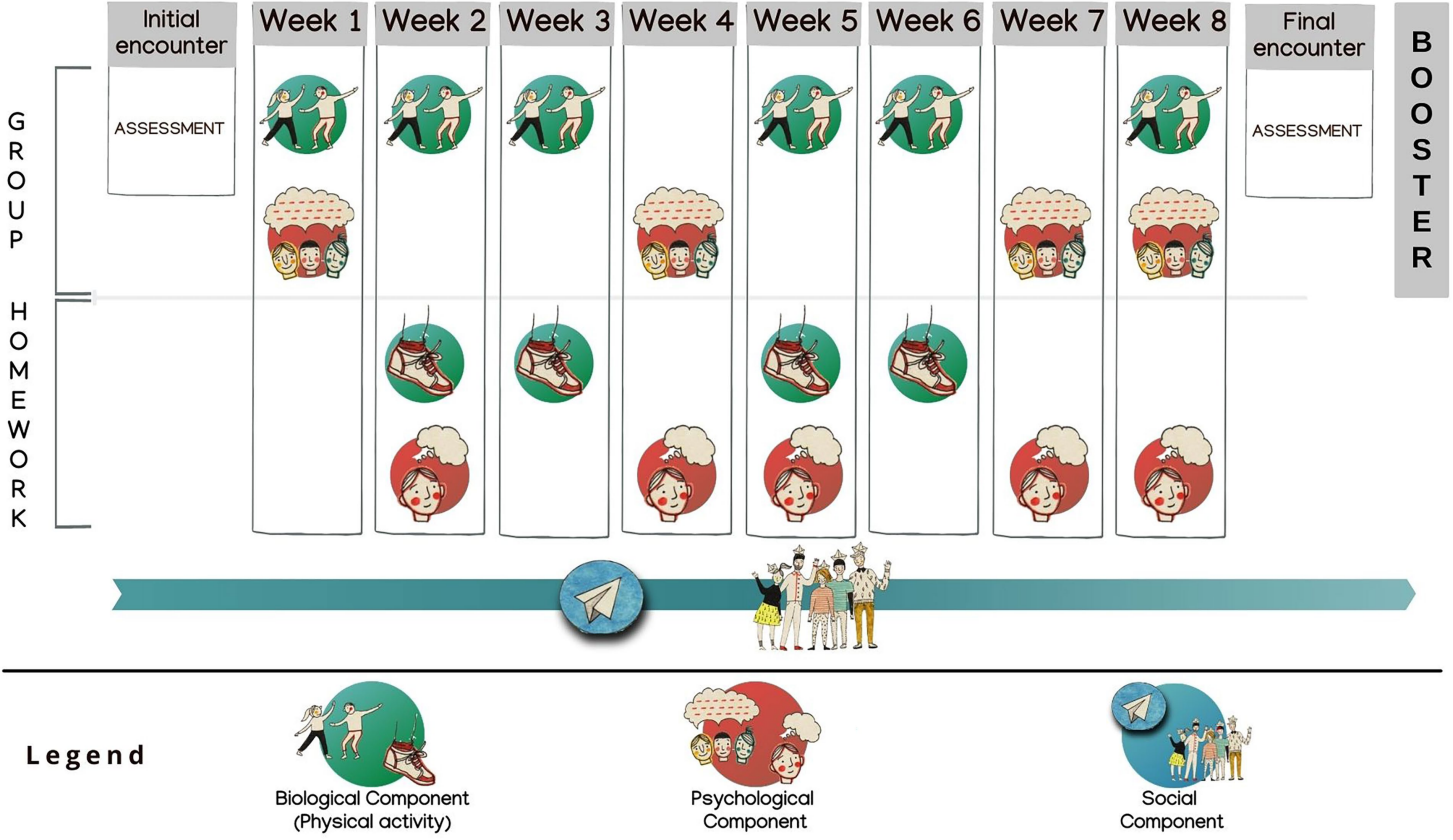
Overview of the ESPRIMO bio-psycho-social intervention.

Considering also the AB suggestions and previous literature recommendation for incorporation of at least one booster session to extend the length of the intervention to a minimum of 3 months in psychological interventions for adolescents and young adults living with chronic illnesses (Sansom-Daly et al., 2012) the intervention has been designed to last 10 weeks with 12 total encounters (one encounter per week except for the first and last week of intervention that have two encounters each), plus one booster session 1 month after the end of the intervention (see Figure 2 for an overview). The booster included a follow-up psychological session which might enhance retention and memory of intervention concepts consolidating what has been practiced during the intervention through a review of the principal constructs (Lochman et al., 2014). Moreover, based on suggestions collected during the focus group, an informal event that welcomed all participants and their beloved followed the formal part of the booster session.

Three different timeframes (lunch break, afternoon, and evening) have been offered to foster participation. Psychological sessions focused mainly on the preferred objectives that emerged in the co-creation; for the physical activity, swing dance sessions were held by trained specialists that could adapt the activities based on the participants’ physical abilities. One psychologist was always present during each session to motivate the participation and to serve as a reference person during the intervention in order to potentially manage the psychological barriers.

Even if contrasting opinions were collected during the different co-creation steps regarding the modalities of the intervention, in-person encounters were chosen given the lifting of preventative measures during the period of the start of the intervention and to account for the desire of human contact after a long period of social distancing. However, personal protective equipment was mandatory during in-person encounters to increase patient safety. Moreover, in addition to the group psychological and physical group face-to-face activities, to foster independence and give the possibility to practice in the most convenient moment of the day (as suggested by participants), YawMS are invited to do some short psychological and physical exercises that were guided by a manual and videos/audios shared on a dedicated Telegram channel. The Telegram channel also allows for informal communication between participants as a space to share thoughts, doubts, or improvements.

All group activities were held in a neutral venue not connected with the health services: a villa managed by a non-profit association that pursues civic, solidarity and social utility purposes by hosting different projects dedicated to the community, young people, and vulnerable people. The building is surrounded by nature, based on participants’ suggestions and the literature on the positive effect of nature on wellbeing (Bratman et al., 2019; Rogerson et al., 2020; Taylor et al., 2022). In order to facilitate accessibility, as suggested by the results of the co-creation, the venue has been selected considering the connection with the city center and its services (e.g., easy parking and disabled access).

### Strengths and limits

To the best of our knowledge, no previous study investigated the needs and preferences of YawMS regarding a biopsychosocial intervention and involved those patients in the co-creation process, making the paper particularly innovative in this field. Moreover, due to the COVID-19 scenario, an adaptation of the methods used for the co-creation process has been implemented, with online modalities for the surveys, FGs and AB discussion. As a further strength, representatives for each psychological, physical, and social domain have been included as stakeholders in the different steps of the co-creation process, together with YawMS representatives. Moreover, a representative of MS patients was also involved in the design of the manual and the revision of this manuscript.

The majority of YawMS included in the surveys and focus groups were female, which, however, is in line with the higher frequency of MS diagnosis in females. The paper presents some limits; a relevant limit is that almost all respondents reported a diagnosis of relapsing–remitting MS, and patients with other types of MS and higher disability were less represented. The preferences and perspectives of those patients should be further explored in future research.

## Conclusion

The co-creation process described in the current paper allowed to integrate evidence from the literature and the clinical expertise of the researchers from the ESPRIMO project with the perspective of the MS main stakeholders (YawMS and HCPs) and to inform the design of the ESPRIMO intervention. Moreover, engaging patients in a meaningful way and fostering an alliance between researchers and patients (emphasizing active involvement, reciprocity, and mutual learning during co-creation) might have empowered patients and reduced the power imbalance between them and researchers.

Preferences and perspectives of YawMS on the relevance, the objectives, modalities, and timing of the intervention but even on strategies to promote participation have been described and might enrich with insights future interventions development and research in the chronicity field. Moreover, the current paper outlined in detail a participative methodology as a model of co-creation in practice which might be replicated in future research on other CDs.

## Data availability statement

The raw data supporting the conclusions of this article will be made available by the authors upon reasonable request.

## Ethics statement

The studies involving human participants were reviewed and approved by the Ethical Committee of the Verona Hospital (Prog 2676CESC). The patients/participants provided their electronic informed consent to participate in this study.

## Author contributions

VD and MR: conceptualization. VD, SP, and MR: data collection and curation. VD, SP, MR, FG, and AG: participant recruitment. SP and VD: formal analysis. SP, MR, and VD: methodology and writing—original draft. AG, MM, FG, VB, LP, and FS: writing—review and editing. All authors contributed to the article and approved the submitted version.

## Funding

Italian Ministry of Research and University (MIUR) 5-year special funding to strengthen and enhance the excellence in research and teaching (Department of Excellence—Dipartimento di Eccellenza). The present study has been partially supported by this programme.

## Conflict of interest

The authors declare that the research was conducted in the absence of any commercial or financial relationships that could be construed as a potential conflict of interest.

## Publisher’s note

All claims expressed in this article are solely those of the authors and do not necessarily represent those of their affiliated organizations, or those of the publisher, the editors and the reviewers. Any product that may be evaluated in this article, or claim that may be made by its manufacturer, is not guaranteed or endorsed by the publisher.
