# Supplementary material for: Promoting participatory research in chronicity: The ESPRIMO biopsychosocial intervention for young adults with multiple sclerosis
Source: Front Psychol. 2022 Nov 3;13:1042234. doi: 10.3389/fpsyg.2022.1042234 (PMC9669711; doi:10.3389/fpsyg.2022.1042234)
Supplement: Supplementary file 1 [file Table_1.DOCX]

Supplementary Material

**APPENDIX 1 - List of the main questions of the survey with young adults with MS**

**The following questions are aimed at understanding how to develop an integrated intervention that takes care of the body and mind at the same time.**

1. In your opinion, how much can your lifestyle affect your disease?

*Not at all* 1 2 3 4 5 6 7 8 9 10 *Much*

1. In your opinion, how important is it that an intervention is based on an integrated vision of mind and body?

*Not important* 1 2 3 4 5 6 7 8 9 10 *Very important*

1. In your opinion, how important is it for an intervention to aim to improve psychological well-being and physical well-being at the same time?

*Not important* 1 2 3 4 5 6 7 8 9 10 *Very important*

1. In your opinion, in order to promote PSYCHOLOGICAL well-being, what are the main objectives on which an intervention should focus (indicate a maximum of 4)?

- It should motivate me to change.
- It should change the way I see things.
- It should inform me about the risks of an unhealthy lifestyle.
- It should help me to accept the disease and its consequences.
- It should provide me with advice and stress management strategies.
- It should make me aware of my emotions.
- It should increase my sense of self-efficacy in managing the disease.
- It should help me to process past traumas.
- It should help me to express my emotions and concerns.

1. Are there any other psychological aspects that you find useful for young people with MS?

- No
- Yes

- If so, which ones?

1. In your opinion, what characteristics should the proposed physical activity have in order to promote PHYSICAL well-being (indicate a maximum of 2)?

- It should be fun.
- It should be adapted to my physical needs.
- It should encourage me to continue with physical activity even after the intervention.
- It should have tangible benefits for the body.
- It should teach me something new.
- It should let me get to know new people.
- It should be a new activity.

1. Are there any other aspects of physical activity that you find useful for young people with MS?

- No
- Yes

- If so, which ones?

1. In your opinion, what would be the ideal frequency for meetings:

- Two meetings per week
- One meeting per week
- One meeting every other week
- One meeting per month

1. In your opinion, what would be the ideal venue for meetings related to psychological aspects:

- My hospital or treatment center
- The seat of a patient association
- A neutral place not connected to my illness (e.g., gym, social club)
- Via telematics (using a videoconferencing platform, e.g., Zoom, skype)

1. In your opinion, what would be the ideal venue for meetings relate to physical activity:

- My hospital or treatment center
- The seat of a patient association
- A neutral place not connected to my illness (e.g., gym, social club)
- Via telematics (using a videoconferencing platform, e.g., Zoom, Skype)

1. If the intervention would be delivered via telematics, what benefits would you see?
2. If the intervention would be delivered via telematics, which critical issues would you see?
3. In general, what could be a barrier for you to take part in these meetings?
4. In general, which strategies could be used to overcome the barrier(s) you have listed?

**APPENDIX 2 - List of the main questions of the survey with healthcare professionals**

**The following questions are aimed at understanding how to develop an integrated intervention that takes care of patient’s body and mind at the same time. Please think about your young patients with MS (age 18-45):**

1. In your opinion, how much can patients’ lifestyle affect their disease?

*Not at all* 1 2 3 4 5 6 7 8 9 10 *Much*

1. In your opinion, how important is it that an intervention is based on an integrated vision of mind and body?

*Not important* 1 2 3 4 5 6 7 8 9 10 *Very important*

1. In your opinion, how important is it that an intervention aims to improve psychological well-being and physical well-being at the same time?

*Not important* 1 2 3 4 5 6 7 8 9 10 *Very important*

1. In your opinion, how much can an integrated biopsychosocial intervention be beneficial for the treatment process?

*Not at all* 1 2 3 4 5 6 7 8 9 10 *Much*

- If you have indicated a score of at least 3, which benefits could it have? _______________

While responding to the following questions, we ask you to:

- refer to young patients with multiple sclerosis (age 18-45 years) with a level of disability at most moderate (Expanded Disability Status Scale (EDSS) score less than 3.5)

- do not dwell on considering the conditions and limitations related to the current pandemic but refer to the usual treatment process

1. In your opinion, in order to promote the PSYCHOLOGICAL well-being of patients, which are the main objectives on which an intervention should focus (indicate a maximum of 4)?

- It should motivate the patient to change.
- It should change the way the patient sees things.
- It should inform about the risks of an unhealthy lifestyle.
- It should help the patient to accept the disease and its consequences.
- It should provide the patient with advice and stress management strategies.
- It should make the patient aware of her/his emotions.
- It should increase patient’s sense of self-efficacy in managing the disease.
- It should help the patient to process past traumas.
- It should help the patient to express her/his emotions and concerns.
- Other aspect(s) _____________

1. In your opinion, what characteristics should the proposed physical activity have in order to promote patients’ PHYSICAL well-being (indicate a maximum of 2)?

- It should be fun.
- It should be adapted to the physical needs of every patient.
- It should encourage the patient to continue with physical activity even after the intervention.
- It should have tangible benefits for the body.
- It should teach something new.
- It should let the patient get to know new persons.
- It should be a new activity.
- Other aspect(s)_______________

1. If the intervention would be delivered via telematics, which benefits would you see?
2. If the intervention would be delivered via telematics, which critical issues would you see?
3. What might be barriers for patients to participate in the meetings?
4. Which strategies could be adopted to resolve or reduce the aforementioned barriers?
5. What might be barriers to proposing to your patients to participate in meetings?
6. Which strategies could be adopted to resolve or reduce the aforementioned barriers?

**APPENDIX 3 – Main characteristics of participants of the surveys**

**3a) Survey with young adults with MS: sociodemographic and clinical characteristics (n=121)**

| **Variables** | **N (%)** |
| --- | --- |
| Gender |  |
| Female | 102 (84) |
| Male | 19 (16) |
| Age |  |
| 18- 27 | 27 (22) |
| 27-36 | 56 (46) |
| 36-45 | 38 (32) |
| Relationship status |  |
| Single | 55 (46) |
| Married/living with a partner | 62 (51) |
| Other | 4 (3) |
| Degree (2 missing) |  |
| No degree | 1 (1) |
| Middle School License | 2 (2) |
| High School Diploma | 61 (51) |
| Undergraduate/Graduate degree | 55 (46) |
| Employment |  |
| Student | 20 (16) |
| Employed | 77 (64) |
| Looking for employment | 11 (9) |
| Other | 13 (11) |
| Diagnosis (1 missing) |  |
| Clinically isolated syndrome (CIS) or radiological isolated syndrome (RIS) | 1 (1) |
| Relapsing-remitting multiple sclerosis (RRMS) | 111 (93) |
| Secondary progressive multiple sclerosis (SPMS) | 4 (3) |
| Primary progressive multiple sclerosis (PPMS) | 4 (3) |
| Time passed since the diagnosis |  |
| Less than a year | 14 (12) |
| 1-2 years | 22 (18) |
| 2-5 years | 37 (31) |
| More than 5 years | 48 (40) |
| Interference of the disease with movements/energy |  |
| It doesn’t limit me at all | 62 (51) |
| It limits me partially | 50 (41) |
| It limits me a lot | 9 (7) |

* Age, μ (σ) = 33 (7)

**3b) Survey with healthcare professionals: sociodemographic and professional characteristics (n=43)**

| **Variables** | **N (%)** |
| --- | --- |
| Gender |  |
| Female | 28 (65) |
| Male | 15 (35) |
| Age* |  |
| 27-40 | 26 (60) |
| 41-53 | 11 (26) |
| 54-66 | 6 (14) |
| Profession |  |
| Physiatrist | 6 (14) |
| Physioterapist | 2 (5) |
| Nurse | 2 (5) |
| Neurologist | 23 (53) |
| Psychologist | 10 (23) |
| Years of experience with SM patients |  |
| Less than 1 year | 3 (7) |
| 1-5 years | 13 (30) |
| 5-10 years | 11 (26) |
| More than 10 years | 1. 37) |

*Age, μ (σ) = 40 (10)
